# Detection and Replication of Moku Virus in Honey Bees and Social Wasps

**DOI:** 10.3390/v12060607

**Published:** 2020-06-02

**Authors:** Andrea Highfield, Jessica Kevill, Gideon Mordecai, Jade Hunt, Summer Henderson, Daniel Sauvard, John Feltwell, Stephen J. Martin, Seirian Sumner, Declan C. Schroeder

**Affiliations:** 1 The Marine Biological Association of the United Kingdom, Citadel Hill, Plymouth PL1 2PB, UK; gidmord@gmail.com (G.M.); jadhun@mba.ac.uk (J.H.); summer.henderson@plymouth.ac.uk (S.H.); 2 School of Environmental and Life Sciences, The University of Salford, Manchester M5 4WT, UK; jkevill@umn.edu (J.K.); s.j.martin@salford.ac.uk (S.J.M.); 3 Veterinary Population Medicine, College of Veterinary Medicine, University of Minnesota, St Paul, MN 55108, USA; 4 Department of Medicine, University of British Columbia, Vancouver, BC V5Z 1M9, Canada; 5 INRAE UR633 Zoologie Forestière, 45075 Orléans, France; Daniel.Sauvard@inrae.fr; 6 Wildlife Matters Consultancy Unit, Battle, East Sussex TN33 9BN, UK; john@wildlifematters.com; 7 Centre for Biodiversity and Environment Research, University College London, Gower Street, London WC1E 6BT, UK; s.sumner@ucl.ac.uk; 8 School of Biological Sciences, University of Reading, Reading RG6 6LA, UK

**Keywords:** honey bee, hornet, wasp, Moku virus

## Abstract

Transmission of honey bee viruses to other insects, and vice versa, has previously been reported and the true ecological importance of this phenomenon is still being realized. Members of the family Vespidae interact with honey bees via predation or through the robbing of brood or honey from colonies, and these activities could result in virus transfer. In this study we screened *Vespa velutina* and *Vespa crabro* collected from Europe and China and also honey bees and *Vespula vulgaris* from the UK for Moku virus (MV), an *Iflavirus* first discovered in the predatory social wasp *Vespula pensylvanica* in Hawaii. MV was found in 71% of *Vespula* *vulgaris* screened and was also detected in UK *Vespa crabro*. Only seven percent of *Vespa velutina* individuals screened were MV-positive and these were exclusively samples from Jersey. Of 69 honey bee colonies screened, 43% tested positive for MV. MV replication was confirmed in *Apis mellifera* and Vespidae species, being most frequently detected in *Vespula* *vulgaris*. MV sequences from the UK were most similar to MV from *Vespula* *pensylvanica* compared to MV from *Vespa velutina* in Belgium. The implications of the transfer of viruses between the Vespidae and honey bees are discussed.

## 1. Introduction

Hornets (*Vespa*) and yellow-jacket wasps (*Dolichovespula* and *Vespula*, the latter abbreviated to *Vs.*) all belong to the family of social wasps, Vespidae, sub-family Vespinae [1]. Nine species of social wasps are found in the UK including eight yellow-jackets and a single hornet species, the European Hornet, *Vespa crabro*. Further to this, the Asian hornet, *Vespa velutina nigrithorax,* has become established in numerous European countries, including Spain, Belgium, and Italy [2,3] after being accidentally introduced into the Bordeaux region of France in 2004 [4]. In the UK the hornet has become established in the Channel Isles and several mature nests have been destroyed on the mainland. It is anticipated that it is only a matter of time until it becomes established on the mainland too [5].

Wasps pose as potential predators to honey bees with yellow-jacket wasps (genera *Vespula*, *Dolichovespula*) preying on dead bees in colonies or those foraging in the field. Yellow-jackets are also known to go into honey bee colonies and rob honey and bee brood [6]; however, the collapse of a honey bee colony due to honey bee-yellow jacket wasp interaction rarely occurs, unless the colony is already weak. Hornets are a more significant predatory threat to honey bees, in particular *V. velutina*, with the threat of this hornet to *Apis mellifera* exacerbated due to European *A. mellifera* having limited defenses against its ‘hawking’ behavior, compared to its Asian counterpart, *A. cerana* [7]. As a consequence of honey bee-wasp interactions, viral exchange is possible, and this could have implications for their populations depending on the pathology and epidemiology of the particular viruses in question [8]. This was demonstrated by Loope et al. [9] who showed that the arrival of the *Varroa* mite on Hawaii resulted in a loss of deformed wing virus (DWV) variant diversity, a virus native to *A. mellifera*, in both honey bees and *Vespula* populations. In honey bee populations this loss in DWV diversity was linked with large scale colony death [10].

DWV has been detected in a range of invertebrates, including bumble bees, wasps, beetles, and wax moths (reviewed by [11]). Several honey bee-associated viruses have also been found in other invertebrates such as Black queen cell virus (BQCV) in *Vespula vulgaris* [12], Acute bee paralysis virus (ABPV), and Kashmir bee virus (KBV) in the German wasp, *Vespula germanica* [13] and DWV and Slow bee paralysis virus (SBPV) in *V. velutina* [14]; however, the interactions of these viruses within social wasps are unknown. There is an ever-increasing list of viruses that have been detected in honey bees with a significant proportion being taxonomically affiliated to the order Picornavirales, including the newly discovered virus, Moku virus (MV). MV was first discovered in the predatory social wasp *Vespula pensylvanica* on Big Island, Hawaii [15]. Next generation sequencing (NGS) data attributed 99.87% of total virus reads derived from the wasp to MV. The high frequency of MV reads detected led the authors to conclude that this species of wasp is likely the native host of MV, although this has not been verified. This study also detected MV-specific reads in honey bee samples and *Varroa* samples from the same locality but at a much smaller depth of sequencing coverage [15]. Since this discovery, Garigliany et al. [14] detected MV in *V. velutina,* from Belgium with a high nucleotide identity (96%) to the Hawaiian MV sequence; however, the abundance of reads from NGS were at a much lower proportion compared to that detected in *Vs. pensylvanica*. Dalmon et al. [16] also found MV sequences in an invasive *V. velutina* collected in France, but they were confined to the gut samples leading the authors to conclude these sequences had originated from ingestion of infected prey.

Indeed, purely the detection of these positive-sense RNA viruses does not necessarily signify that they are actively replicating in these hosts, although, for an RNA virus to dominate an NGS sequencing library to such an extent makes replication the most plausible explanation. Screening for the negative sense RNA of the virus can confirm that active replication is taking place; however, this can be challenging in itself due to the tendency of reverse transcriptase to false priming events. To overcome these problems, a non-homologous tag sequence can be added to the primer used in the reverse-transcription with the subsequent PCR then using this tag sequence as a primer in combination with the other target primer. Reverse transcription can also be undertaken using a thermostable reverse transcriptase at elevated temperatures to further reduce the likelihood of false priming. The resultant cDNA can then be treated with exonuclease in order to eliminate the primer that was used for the reverse transcription participating in the PCR, thereby further reducing the chance of false positives [17]. To our knowledge, the only study to date to have shown replication of honey bee viruses in the Vespidae is Yanez et al. [18] who detected the negative sense RNA of Israeli acute paralysis virus (IAPV) in *V. velutina*.

Following on from previous observations of MV in invasive *V. velutina* in Europe [14,16], we set out to extend on these studies by performing an exploratory preliminary screen of MV in Asian and European hornets collected in Europe and China. We also extended our screening to the UK honey bee population and also the common wasp, *Vs. vulgaris*. Finally, to establish whether active viral replication is occurring in the different species, and the extent of virus transfer, we developed an assay to detect the replicative form of MV.

## 2. Materials and Methods

Thirty honey bees from each of the sixty-nine honey bee colonies were sampled across England and Wales, the majority between April and October 2016 [19], with three colonies sampled in October 2015 and one in March 2017 (Supplementary Materials Table S1).

Six *V. crabro* (three from a colony located in Henley Down, Sussex, UK and three from Saint-Martial, France) were collected between 30 August and 5 September 2016. Six *Vs. vulgaris* were analyzed that had been collected from traps located in Stoke-on-Trent, Lewes, Gloucester, Bournemouth, Owestry and Rye as part of the UK-based Big Wasp Survey in 2017 [20]. A further twelve adult *Vs. vulgaris* were processed that had been sampled from three wasp colonies collected from Dorchester and Basingstoke, UK along with three larvae sampled from the Dorchester colony in October 2017 (Supplementary Materials Table S1).

Twenty-three invasive *V. velutina* (two from Orléans; nine from Saint-Hilaire-Saint-Mesmin; twelve from Saint-Jean-le-Blanc, France), were collected between 28 September–7 October 2016, and three were collected from a nest located on the island of Jersey in October 2018. Four native *V. velutina* individuals from China were also processed that had been collected from nests located in Hangzhou and Nanjing in 2018 (Supplementary Materials Table S1).

Individual wasps/hornets were ground up in liquid nitrogen to a fine powder. An amount of 30 mg tissue powder was used for RNA extraction using the Macherey-Nagel Nucleospin RNA extraction kit according to manufacturer’s instructions, with the exception of the elution volume, which was 40 µL. Honey bee samples were processed according to Highfield et al. [21] with the exception of pools of 30 bees per colony that were processed. Primer sets used in this study can be found in Table 1.

For detection of the genomic RNA of MV, fifty nanograms of extracted RNA was used in One-step RT-PCR reactions using the One-step Sensifast kit (Bioline) according to manufacturer’s recommendations using MVF and R primers to amplify a 140 bp fragment (Table 1), which spans domain I and II of the RNA dependent RNA polymerase (RdRp). Reactions proceeded with an initial incubation at 45 °C for 10 min, 95 °C for 10 min, followed by 40 cycles of 95°C for 15 s, 54 °C for 15 s and 72 °C for 15 s. A selection of samples that were MV positive were also screened for negative sense MV (Supplementary Materials Figure S1) (8 × *A. mellifera* samples, 7 × *Vs. vulgaris,* 3 × *V. crabro* and 2 × *V. velutina*). For the detection of negative sense MV, 500 ng RNA was reverse transcribed with Superscript IV (Invitrogen) using the tagMV primer at 55 °C [22]. Synthesized cDNA was then treated with ExosapIT (Thermo Scientific) following manufacturer’s instructions. An amount of 1 µL exonuclease-treated cDNA was PCR-amplified using the Sensifast kit (Bioline) and primers PCRtagF and MVR. Reactions were undertaken in 10-µL volumes and were incubated at 95 °C for 2 min, followed by 40 cycles at 95 °C for 5 s, 57 °C for 10 s, and 72 °C for 10 s. Post-PCR melt curve analysis and agarose gel electrophoresis were undertaken to confirm the presence of the correct MV product. Selected PCR products were sequenced by Source Bioscience (Cambridge, UK) and are deposited under accession numbers MK829246-MK829256 and MT251349-MT251361. BioEdit v7.2.6 was used to construct a multiple sequence alignment of MV sequences generated in the study, and the tree was constructed using PhyML [23] with 100 bootstraps. The alignment and tree were visualized using the ggtree package within R [24].

## 3. Results

**Figure 1:**
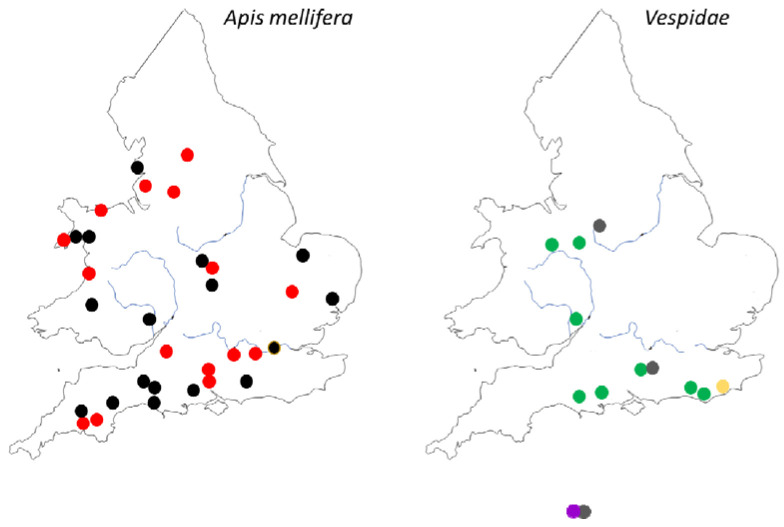
Presence of Moku virus (MV) in England, Wales, and Jersey, where red dots are locations of *A. mellifera* MV-positive colonies and black are MV-negative (where multiple colonies were sampled from the same site, detection in 1+ colonies have been displayed as positive). Yellow is the location of MV-positive *V. crabro,* green is MV-positive *V*s. *vulgaris*, purple is MV-positive *V. velutina* (Jersey), and grey is the locations of MV-negative vespid samples.

MV was not detected in any of the *V. velutina* individuals collected from France or China. The only *V. velutina* individuals that were positive for MV were sampled from Jersey. The UK *V. crabro* collected in Sussex (Figure 1), were positive for MV yet the other three *V. crabro* collected in France were all below the limit of detection for MV.

Of the fifteen adult *Vs. vulgaris* individuals that were screened, ten were MV-positive from locations detailed in Figure 1, with MV-free individuals sampled from colonies in Basingstoke and a wasp trap in Stoke-on-Trent. A further three out of three *Vs. vulgaris* larvae from a colony in Dorchester were also analyzed and were positive for the virus. MV was detected in the UK honey bee population in thirty of the sixty-nine samples across a broad geographic range (Figure 1). The samples were primarily collected during the months of May to October, with those collected later in the year being more likely to have MV. Of twelve colonies sampled in October, 100% were positive for MV, compared to May where eleven colonies were sampled and only 9% were positive (Supplementary Materials Table S1). In the preceding months, 50% of samples collected in September and 12.5% in April were MV-positive, indicating an increasing trend of MV incidence later in the season.

Occurrence of the replicative form of MV was lower but was confirmed in *A. mellifera*, *V. velutina*, and *Vs. vulgaris*. Seventy-one percent of seven *Vs. vulgaris* individuals analyzed were positive for MV antisense, including brood. The replicative form of MV was only detected in one out of eight honey bee colonies that had previously tested positive for genomic MV. One of two MV positive Jersey *V. velutina* were also confirmed to have the MV negative-sense, but none of the *V. crabro* had a signal for MV antisense.

**Figure 2:**
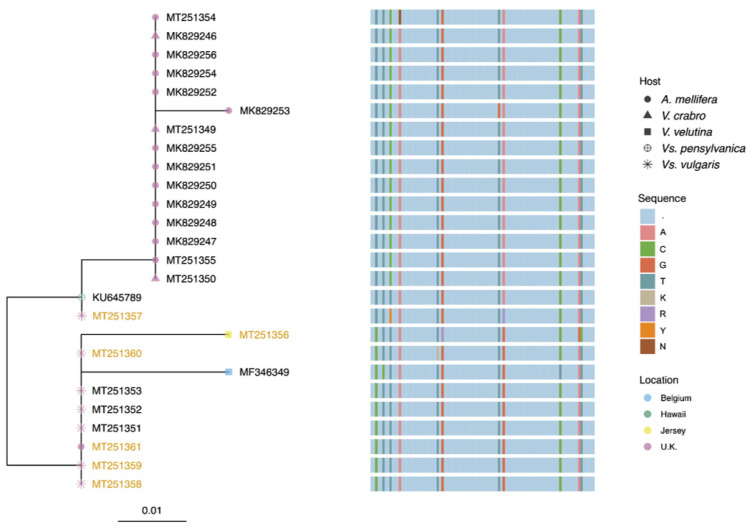
Phylogenetic tree and multiple sequence alignment of Moku virus sequences based on a region of the RdRp (8801 to 8894 nt on MV KU645789.1). Branch tip-points are colored by the location, and shaped by host. Tip labels that are colored yellow are derived from the negative sense assay. A multiple sequence alignment is shown next to corresponding sequences. Skyblue represent identical nucleotide sequences compared to the consensus, and the colored bars represent base pair substitutions.

Ten of the twelve honey bee MV sequences had 99% identity to the MV sequence detected in *Vs. pensylvanica* on Big Island, Hawaii and with 97% identity to the *V. velutina* sequence from Belgium. One of the two other honey bee MV sequences (accession number MK829253) had a single nucleotide substitution compared to the Hawaiian MV genotype, accounting for 98% identity; and 94% identity to MV in *V. velutina* from Belgium (Figure 2). The remaining honey bee-derived MV sequence had one ambiguous nucleotide, most likely due to multiple sequences present. The sequences from the *V. crabro* individuals also shared 99% identity to the Hawaiian genotype. All the MV sequences from *Vs. vulgaris* shared 98% similarity with both the Hawaiian and the Belgium genotypes but with nucleotide substitutions at different positions. The MV sequence from *V. velutina* sampled in Jersey was most similar to the sequences from *Vs. vulgaris*. The majority of sequences from the negative sense assay for *Vs. vulgaris* were identical to the sequences from the standard assay with the exception of 3136 (MT251357). This had ambiguous bases at two positions, again, most likely due to the presence of multiple variants in the individual analyzed. The replicative form in *A. mellifera* was identical to the MV strain in *Vs. vulgaris*. Unfortunately, the partial MV sequence detected by Dalmon et al. [16] in the gut of *V. velutina* does not encompass the region of the RdRp used in this study therefore we could not include it in the analysis. Aside from the ambiguous nucleotides, which were likely a result of more than one sequence present and thus the amino acid could not be determined, only two of the nucleotide substitutions manifested in an amino acid substitution. These substitutions were detected in the honey bee sample FTN, and the *V. velutina* from Jersey.

## 4. Discussion

MV was only detected in *V. crabro* individuals from the UK but not in those collected from France. MV was detected in *V. velutina* from Jersey but not in the twenty-three *V. velutina* individuals from France or the samples from China, although MV has previously been reported for this species in Belgium and France [14,16]. MV was detected in the majority of *Vs. vulgaris* samples analyzed. In the honey bee colonies screened across England and Wales, MV was frequently detected indicating that this virus has been at least circulating in UK *A. mellifera*, *Vs. vulgaris* and *V. crabro* populations since 2016. Given that the first sighting of *V. velutina* in the UK was only reported in 2016, the same year as the majority of the honey bee samples were collected, it is unlikely that *V. velutina* is the source of MV introduction into the UK, due to it already being widespread in UK honey bees. If MV is truly native to the *Vespula* species, as proposed by Mordecai et al. [15], we can surmise that MV was introduced into UK honey bees from *native* UK vespid populations.

Dalmon et al. [16] concluded that MV detected in *V. velutina* in France were likely to have originated from MV-infected prey, as the virus was exclusively detected in the gut of an individual that was analyzed. To verify whether MV is replicating in the species analyzed in this study, the negative sense assay was employed and did indeed confirm replication in all species with the exception of *V. crabro*; however, as such a low sample size was analyzed we cannot ascertain whether MV definitely cannot replicate in this species. In fact, as the MV *V. velutina* sequence from Jersey was most similar to the mainland UK *Vs. vulgaris* sequences we hypothesize that invasive *V. velutina* in Jersey could have acquired the virus from native *Vs. vulgaris.* Phylogenetically, *Vs. vulgaris* and *Vs. pensylvanica* form a monophyletic group along with *Vs. flaviceps*, *Vs. germanica*, *Vs. maculifrons*, *Vs. alascensis,* and *Vs. flavopilosa* [25]. It is therefore conceivable that the natural host range of MV is across this clade and that the lower levels of detection in non-*Vespula* species are actually a result of acquisition through predator/prey interactions or spillover.

The pathological effects of virus infection in honey bees can be obvious, such as emerging honey bees with wing deformities due to infection by DWV. Honey bee viruses can also persist at high levels in individuals with no apparent symptoms of disease [21]; however, less discernable impacts of virus infection in honey bees are known to exist, such as reduction in life expectancy and foraging success [26]. Presently, the pathological effects of MV on honey bees and wasps is unknown and it is unclear how long MV has been circulating. As a result, the impact of this virus on honey bee and wasp populations cannot be gauged; however, the discovery of its replication in honey bees flags it up as an entity that warrants further investigation. Whilst the incidence of the replicative form in honey bees was very low, we do not know enough about the seasonal fluctuations in MV occurrence to ascertain whether this could just be an artifact of sampling. Interestingly, incidence of MV, as assessed by the standard assay, was higher later in the season, potentially indicating some seasonality of virus incidence. A more detailed analysis of MV loads in different hosts to further understand population dynamics of this virus and the mechanisms of viral emergence is required to confirm this.

Multiple variants of MV were detected in this study as seen with various other honey bee/insect viruses, demonstrating that MV exists as a viral quasispecies. The sequences derived for MV from *V. crabro* and UK honey bees were more similar to the sequence from *Vs. pensylvanica* on Big Island, Hawaii, compared to geographically closer Belgium MV sequences from *V. velutina*. The MV sequences from UK *Vs. vulgaris* samples were different to other UK sequences found in the other species (with the exception of the replicative MV in *A. mellifera*). This could suggest that MV variants circulating have some host specificity; however, more data would be required to support this hypothesis. This may be true in other virus/host systems with, *Aphid lethal paralysis virus* (ALPV), a virus originally characterized as an infectious agent of the aphid, *Rhopalosiphum padi* [27], existing as a viral quasispecies, with two novel ALPV variants reported from non-aphid insect hosts, as well as a variant presently only found in honey bees [28].

The MV sequence detected in *A. mellifera* using the standard screening assay was not the same as the sequence detected in the negative sense assay, which was identical to the *Vs. vulgaris* MV sequence. Whist more data is required to verify, this could indicate that only certain variants are able to replicate in multiple hosts or it could be a limitation of Sanger sequencing, which is not able to capture the full diversity of a viral quasispecies. One sequence detected in the standard assay from a honey bee sample and also the *V. velutina* Jersey sequence did have an amino acid substitution showing that nucleotide substitutions may manifest in changes in protein sequence which could ultimately have an effect on viral epidemiology.

This study illustrates the complexity of these RNA virus systems, with MV potentially being transmitted between honey bees, and the Vespidae. Whilst the different species may only be carriers of various RNA viruses, ultimately the virus could adapt to new hosts and pathological effects may emerge. Increases in the number of cross species viral transmission routes, such as from the introduction of invasive species, raises the possibility of newly mutated forms re-emerging. We have highlighted the importance of the Vespidae as another factor within the RNA virus matrix that circulates in and around honey bees. With continually mutating virus quasispecies, new emerging infections are always possible, especially when they are crossing the species boundary. The challenge for the honey bee community is anticipating and limiting such outbreaks.

## Figures and Tables

**Table 1:** Primers used in this study. Bold nucleotides indicate the tag sequence.

Primer	Purpose	Primer Sequence (5′-3′)
MVF	One-step RT-PCR of Moku virus (MV)	GACTGTTTAAAGGATTACCG
MVR		GCACCTCTATAAGCAGAGAG
tagMV	Reverse transcription of negative sense MV	**GGCCGTCATGGTGGCGAATAA**GACTGTTTAAAGGATTACCG
PCRtagF	PCR of negative sense MV	GGCCGTCATGGTGGCGAATAA

## Supplementary Materials

The following are available online at https://www.mdpi.com/1999-4915/12/6/607/s1, Figure S1: RT-PCR detection of MV and strand-specific RT-PCR of replicative MV, Table S1: Samples analyzed in this study.
