# Supplementary figures and images for: Detection and Replication of Moku Virus in Honey Bees and Social Wasps
Source: Viruses. 2020 Jun 2;12(6):607. doi: 10.3390/v12060607 (PMC7354477; doi:10.3390/v12060607)

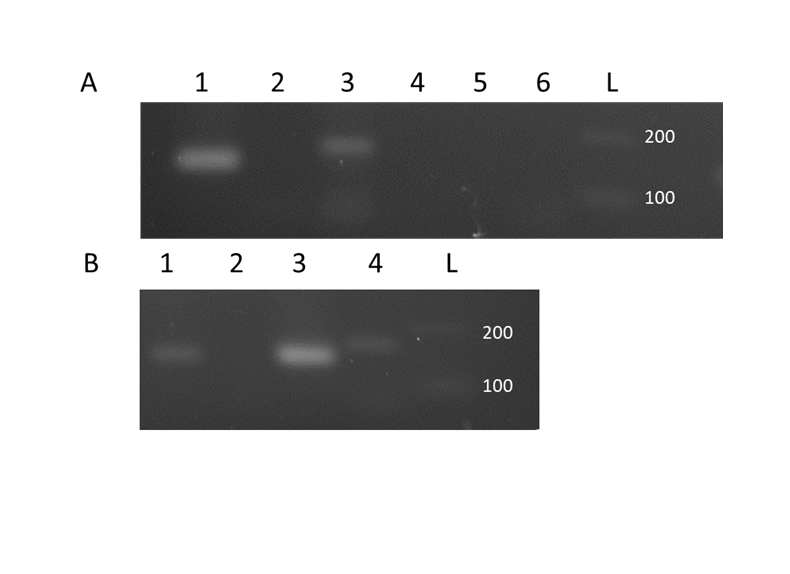

Supplement: Supplementary file 1 [file viruses-12-00607-s001.zip › Supp Fig 1 gel .tif]
